# Exploration of mycovirus composition in a hypovirulent strain of *Sclerotinia sclerotiorum* potentially uncovers mycovirus cross-taxa transmission

**DOI:** 10.1016/j.virusres.2025.199552

**Published:** 2025-02-26

**Authors:** Lixia Gao, Weimeng Li, Jichun Jia, Jiasen Cheng, Yanping Fu, Xueqiong Xiao, Qing Cai, Yang Lin, Tao Chen, Bo Li, Xiao Yu, Tom Hsiang, Daohong Jiang, Jiatao Xie

**Affiliations:** a National Key Laboratory of Agricultural Microbiology, Huazhong Agricultural University, Wuhan, China; b Hubei Key Laboratory of Plant Pathology, College of Plant Science and Technology, Huazhong Agricultural University, Wuhan, China; c Hubei Hongshan Laboratory, Wuhan, China; d College of Plant Protection, Laboratory of Integrated Pest Management in Agriculture, Shanxi Agricultural University, Jinzhong, Shanxi, China; e Environmental Sciences, University of Guelph, Guelph, Ontario, Canada

**Keywords:** Mycoviruses, Co-infections, *Sclerotinia sclerotiorum*, Hypovirulence

## Abstract

*Sclerotinia sclerotiorum* is a worldwide plant pathogenic fungus. Identifying novel mycoviruses in this fungus can aid in developing fungal disease control strategies and enhance our understanding of viral evolution. Here, we analyzed mycovirus composition in *S. sclerotiorum* strain XZ69, and identified six ssRNA mycoviruses, including five known mycoviruses and one unassigned mycovirus. The newly identified mycovirus, tentatively named Sclerotinia sclerotiorum narna-like virus 1 (SsNLV1/XZ69), possesses a full-length genome of 3534 nucleotides, containing a single ORF that encodes an RNA-dependent RNA polymerase (RdRp) of 1090 amino acids. The RdRp encoded by SsNLV1/XZ69 shares 60.4 % identity with that encoded by Monilinia narnavirus H. SsNLV1/XZ69 phylogenetically clusters with unclassified narna-like viruses potentially infecting fungi, plants, and animals, and they form an independent branch that is distant from established families, therefore supporting the establishment of a new family to accommodate these viruses. Sclerotinia sclerotiorum fusarivirus 3 (SsFV3/XZ69) share 97 % amino acid identities with preciously reported Botrytis cinerea fusarivirus 8 (BcFV8). This last mycovirus originated from *Botrytis cinerea*, and hence this reveals that cross-genus transmission of SsFV3 or BcFV8 between *B. cinerea* and *S. sclerotiorum* may have potentially occurred. Mycovirus elimination, horizontal transmission, and RNA transfection experiments revealed that Sclerotinia sclerotiorum negative-stranded RNA virus 1 (SsNSRV1/XZ69), SsNSRV2/XZ69, and SsFV3/XZ69 may be associated with hypovirulence in *S. sclerotiorum*, and strain XZ69 exhibits potential disease biocontrol on rapeseed seedlings. Our study expands our understanding of viral evolution, and may provide new potential biocontrol agents for *S. sclerotiorum*.

## Highlights

• The strain XZ69 of *Sclerotinia sclerotiorum* is co-infected by six ssRNA mycoviruses.
• SsNLV1 is phylogenetically related to viruses infecting organisms across three kingdoms.
• SsFV3 could potentially occur cross-genus transmission between *B. cinerea* and *S. sclerotiorum*.
• Strain XZ69 exhibits potential for rot disease biocontrol on rapeseed seedlings.

## 1 Introduction

Viruses, including mycoviruses, are ubiquitous and play crucial roles in ecosystems. Mycoviruses can cause latent infections, as well as positively or negatively regulate virulence in phytopathogenic fungi (Ghabrial et al., 2015; Roossinck, 2019). Mycoviruses conferring hypovirulence have been gaining attention due to their potential for development of biocontrol agents against plant fungal diseases. With the advance of next-generation sequencing technologies, the discovery of mycovirus quantity has surged, greatly enriching our understanding of mycoviral diversity in terms of genome organization, lifestyle, replication cycle, evolution, and transmission (Ayllón and Vainio, 2023; Xie and Jiang, 2024).

The co-infection by multiple mycoviruses in a single host strain results in complexity and diversity. For instance, *Magnaporthe oryzae* strain YC81–2 contains six mycoviruses (Liu et al., 2020). *Phytophthora condilina* strain BD661 is co-infected with 15 mycoviruses (Botella and Jung, 2021). High-throughput sequencing has identified the co-infection of *Fusarium poae* strain (MAFF 240,374) with 16 mycoviruses (Osaki et al., 2016), *Sclerotium Rolfsii* strain BLH-1 with 21 mycoviruses (Zhu et al., 2018). Additionally, the interactions of mycoviruses and hosts can affect host phenotypes, including synergistic (Sun et al., 2006), antagonistic (Chiba and Suzuki, 2015), mutualistic relationships (Zhang et al., 2016), and mycovirus genome rearrangements (Sun and Suzuki, 2008). Like the synergistic effect that exists between Cryphonectria hypovirus 1 (CHV1) and mycoreovirus 1 in *Cryphonectria parasitica* (Sun et al., 2006). Such interactions show promise for disease biocontrol in agriculture (Kondo et al., 2022; Rigling and Prospero, 2018; Yu et al., 2010).

The 2024 update of the USDA Fungal Database (https://fungi.ars.usda.gov/) includes 1435 records related to *S. sclerotiorum*. A closer examination of these records shows that *S. sclerotiorum* can be found on more than 700 species of plants, primarily dicotyledonous crops and numerous weeds, and these are distributed all over the world*.* Sclerotinia stem rot caused by *S. sclerotiorum* results in yield loss and quality reduction of rapeseed (Shang et al., 2024; Sharma et al., 2018). Interestingly, *S. sclerotiorum*, is also found as a beneficial fungus for gramineous crops, can also grow endophytically to provide resistance against fungal pathogen infections and promote growth in gramineous plants, including wheat and rice (Tian et al., 2020). Mycoviruses in *S. sclerotiorum* are very common and harbor complex genomic types with dsRNA, -ssRNA, +ssRNA, and ssDNA genomes. More importantly, some of them offer great potential for biological control due to their mediated hypovirulent characteristics in *S. sclerotiorum*. For instance, two hypovirulence-associated mycoviruses (HAMV), Sclerotinia sclerotiorum mycoreovirus 4 (SsMyRV4) and Sclerotinia sclerotiorum hypovirulence-associated DNA virus 1 (SsHADV1), exhibit biocontrol potential against Sclerotinia stem rot in different ways. SsMyRV4 can promote horizontal transmission efficiency of heterologous HAMV between incompatible individuals of *S. sclerotiorum* by inhibition of host non-self recognition (Wu et al., 2017). SsHADV1 can convert the phytopathogenic fungus *S. sclerotiorum* into a beneficial endophytic fungus in rapeseed plants (Zhang et al., 2020) and by inducing basic plant resistance to *S. sclerotiorum* infection, and by spreading in *S. sclerotiorum* populations in rapeseed fields via hyphal fusion (Xie and Jiang, 2024). In addition, some mycoviruses in *S. sclerotiorum* show unique evolutionary lineages and enrich genetic diversity. For example, the discovery of Sclerotinia sclerotiorum deltaflexivirus 1 (SsDFV1) has led to the proposal of establishing a new family, *Deltaflexiviridae* (Li et al., 2016). Similarly, the identification and discovery of Sclerotinia sclerotiorum negative-stranded RNA virus 1 (SsNSRV1) have led to the establishment of *Mymonaviridae*, which has been recognized and included by the International Committee on Taxonomy of Viruses (ICTV) (Liu et al., 2014). These taxonomic proposals reflect the genetic and evolutionary diversity observed within mycoviruses infecting *S. sclerotiorum* and highlight the need for a more nuanced classification system to accommodate the unique characteristics of these mycoviruses.

In the present study, we explored mycovirus composition in the hypovirulent strain XZ69, and determined the full-length cDNA sequences of two mycoviruses, including a novel narna-like virus and a previously reported virus. We also assessed the biocontrol potential of strain XZ69 and elucidated the role of mycoviruses in hypovirulence of *S. sclerotiorum*. This study provides new suggestions for the evolution of narna-like viruses, and identified a new mycovirus-mediated hypovirulent strain as a biocontrol resource against stem rot caused by *S. sclerotiorum*.

## 2 Materials and methods

### 2.1 Fungal strains and culture conditions

The *S. sclerotiorum* strain XZ69 was isolated from a single sclerotium collected from diseased rapeseed stems at Linzhi City (94°E, 29°N), Tibet, China. This strain was obtained as follows: Sclerotia were surface-disinfected with 75 % alcohol for 1 min, then washed three times with sterilized water. The dried sclerotia were cultured on potato dextrose agar (PDA) at 20 °C for 3 days. The fresh mycelia from sclerotia were sub-cultured several times onto PDA to obtain strain XZ69. Strain 1980 is a virulent strain of *S. sclerotiorum* that has been subjected to genomic sequencing which is available on NCBI (ASM14694v2). Strain 1980R was labeled with a hygromycin resistance gene (Hai et al., 2024).

### 2.2 Biological characterization of *S. sclerotiorum* strains

To assess the biological traits of strain XZ69, the growth rate and virulence were measured as previously described with slight modifications (Luo et al., 2022). Each treatment was repeated at least three times, and all experiments were conducted in triplicate. Growth rate was recorded by measuring colony diameter at 24 and 48 h post-inoculation (hpi), and colony morphology on PDA was photographed at 7 days post-inoculation (dpi). Experimental data were analyzed and visualized using GraphPad Prism 8. Statistical significance between means was determined using the *t*-test method, with significance defined at *p* < 0.05.

### 2.3 Total RNA extraction and sequencing

We conducted metatranscriptomic analysis to explore mycovirus composition in strain XZ69. The strain XZ69 was cultured on cellophane-layered PDA for 4 days. One gram of mycelium was collected and ground into a fine powder in liquid nitrogen. Total RNA was extracted using a Trizol RNA extraction kit (TaKaRa, Dalian, China), and treated with DNase I (TaKaRa, Dalian, China), and quantified using a NanoDrop 2000 spectrophotometer (ThermoFisher Scientific, Waltham, MA, USA). After removing rRNA from the total RNA, the samples undergo fragmentation, reverse transcription, purification, end repair, and adapter ligation. Library quality was assessed using Qubit 2.0 and Agilent 2100, followed by Q-PCR for accurate quantification, yielding a strand-specific library. The rRNA-depleted RNA-Seq library was constructed using the Nova-PE150 strategy by Novogene Company, Beijing. The cDNA library was sequenced on the HiSeq 2500 platform (Illumina, USA), generating 100,170,130 raw paired-end (150 bp) reads. Using Trimmomatic (v0.36) (Bolger et al., 2014), adapter sequences and low-quality reads were removed, leaving 99,792,750 high-quality reads. These reads were mapped to the *S. sclerotiorum* genome (GCF_000146945.2) via Bowtie (v2.0) (Langmead and Salzberg, 2012), and 4912,932 unmapped reads were *de novo* assembled using Trinity (v2.5.0) (Haas et al., 2013) and SPAdes (v3.11.1) (Bankevich et al., 2012), yielding 14,244 contigs. BLASTx analysis revealed that only 0.2 % (24/14,244) of contigs were virus-related. Contigs >1000 bp were compared against the NCBI non-redundant protein database (E-value ≤10^–5^), and viral sequences were identified using DIAMOND BLASTx. Potential ORFs were analyzed with DNAMAN 8, and virus-related sequences were assembled and annotated as previously described (Jia et al., 2021). The metagenomic data are available in the SRA database under accession number PRJNA1193795.

### 2.4 Mycovirus detection and terminal sequence determination

To verify the contigs obtained by RNA-Seq, cDNA was synthesized using a reverse transcription kit (TaKaRa, Dalian, China). Specific primers were designed from sequenced virus-related contigs to detect mycoviruses in strain XZ69 via PCR amplification, and the amplicons sequenced. The terminal sequences of mycovirus genomes were obtained using rapid amplification of cDNA ends (RACE) technology, and the RACE was conducted twice (Scotto-Lavino et al., 2006). The primers used for viral detection and RACE are listed in Table S1.

### 2.5 Phylogenetic analysis

To evaluate the evolutionary relationship of these mycoviruses, RdRp sequences encoded by the mycoviruses were obtained by BLASTp against NCBI database. DNAMAN 8 software was used to analyze the basic characteristics of the viral genome and potential gene ORFs. Motifs were predicted at this website (www.genome.jp/tools/motif/). Jalview2.11.4.0 software was used for multiple sequence alignment of the RdRp domain, and virus information is listed in Table S2. A phylogenetic tree based on RdRp aligned sequences was constructed using PhyloSuite1.2.2, where the MAFFT module was used for multiple sequence alignment, the trimAL module was used for trimming and aligning sequences, and the IQ-TREE module was used to construct an ML phylogenetic tree with automatic model selection based on 1000 bootstrap replicates (Zhang et al., 2020). FigTree v1.4.4 was used for the display and editing of phylogenetic trees.

### 2.6 Horizontal transmission, elimination, and RNA transfection

To define the contribution of mycoviruses to the hypovirulent traits of strain XZ69, we conducted three experiments, including mycovirus horizontal transmission via hyphal fusion, elimination by single-protoplast isolation, and PEG-mediated mycovirus RNA transfection.

For horizontal transmission of the six mycoviruses infecting strain XZ69, the strain was dual-cultured with strain 1980R (recipient) for 3 days on PDA. New isolates were picked from the edge of the colony of 1980R, and transferred to a new PDA containing 50 μg/mL hygromycin B. The newly obtained strains were subcultured three times onto PDA amended with hygromycin B, and then their phenotypes observed. For mycovirus elimination, protoplasts of strain XZ69 were prepared as described previously (Zhang et al., 2009) and were regenerated on RM (regeneration medium: 0.7 M sucrose, 0.5 g/L yeast extract, 10 g/L agar) containing 50 ng/mL ribavirin and incubated for 3 days. The protoplast regenerated strains were picked out and then sub-cultured three times on PDA. Additionally, to explore whether genomes of three mycoviruses had transfected the protoplasts of strain 1980R in the presence of polyethylene glycol (PEG), total RNA of strain XZ69-D4 co-infected by SsNRSV2/XZ69, SsFV3/XZ69, and SsNLV1/XZ69 was extracted. A brief protocol is as follows: 20 µg of total RNA of strain XZ69-D4 was mixed with 50 µL of protoplasts (10^7^ /mL) of strain 1980R and incubated on ice for 30 min. Subsequently, 800 µL of PEG 4000 (60 %, w/v) was mixed uniformly with 400 µL of sterile KTC solution (1.8 M KCl, 150 mM Tris–HCl pH=8.0, 150 mM CaCl_2_) to prepare the protoplast transformation solution, and 1.2 mL of protoplast transformation solution in the aforementioned protoplasts were incubated at 20 °C for 20 min. The solution was then spread evenly onto RM medium. Hyphae from RM medium were transferred to fresh PDA containing 50 μg/mL hygromycin B and subcultured three times.

When the phenotypes of newly obtained strains by horizontal transmission or single-protoplast isolation or transfection were stable, the mycovirus presence was assessed by RT-PCR with mycovirus-specific primers (Table S1).

### 2.7 Biological control assay

To verify the biocontrol potential of strain XZ69, we treated rapeseed seedlings with a mycelial suspension of strain XZ69, which was prepared as follows. Each fresh mycelia of *S. sclerotiorum* strains XZ69 and 1980 were added to 100 mL of PDB (potato dextrose broth) and incubated with shaking (130 rpm) at 20 °C for 2 days. The mycelia were filtered out with Miracloth (Merck, Germany), and equal amounts of mycelium were weighed out (2 g), and then 20 mL of fresh PDB was added. The mycelium was chopped in a blender up to 5 s and transferred into a 50 mL conical flask. It was then incubated on a shaker (130 rpm) at 20 °C for 1 h. We pre-sprayed rapeseed seedlings with equal amounts of fresh mycelial suspensions of strains 1980 or XZ69, followed by a secondary spray of mycelial suspension of strain 1980 after 24 h. Photographs were taken 48 h after the second spray.

## 3 Results

### 3.1 Biological characteristics of *S. sclerotiorum* strain XZ69

**Fig. 1:**
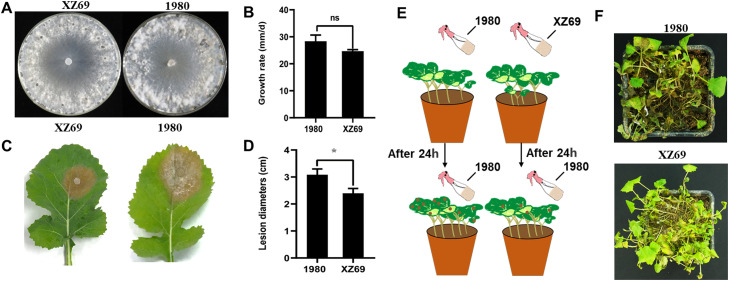
The biological characteristics of *S. sclerotiorum* strain XZ69 and 1980. (A) Colony morphology of hypovirulent strain XZ69 and virulent strain 1980 on PDA at 20 °C for 7 days. (B) Determination and analysis of the growth rate of hypovirulent strain XZ69 and virulent strain 1980 on PDA at 20 °C for 48 h. Error bars representing the standard deviation calculated from three replicates. Significant differences at a confidence level of *p* < 0.05 were determined using *t*-test. (C) Collection of photographs of lesions formed by fresh mycelium of hypovirulent XZ69 and virulent strain 1980 inoculated on detached rapeseed leaflets at the 4–5 leaf stage cultivated on PDA and collection of lesion diameter data at 48 h post-inoculation. (D) Data analysis of lesion diameters formed by fresh mycelium of hypovirulent strain XZ69 and virulent strain 1980 inoculated on detached rapeseed leaflets at the 4–5 leaf stage. Error bars representing the standard deviation calculated from three replicates. Significant differences at a confidence level of *p* < 0.05 were determined using *t*-test. (E) Schematic diagram of biological control. (F) Biological control efficacy of strain XZ69.

To clarify whether strain XZ69 has potential to develop a biocontrol agent, the pathogenicity, growth rate and biocontrol effect of this strain were determined. Compared to the virulent strain 1980 of *S. sclerotiorum*, strain XZ69 exhibited relatively thinner hyphal growth at 2 dpi on PDA, but no significant differences in colony morphology were observed at 7 dpi, where strain XZ69 displayed normal colony morphology (Fig. 1A). The growth rate of strain XZ69 was 2.47 cm/d, which was not significantly different than that of strain 1980 (2.83 cm/d) (Fig. 1B). Both strains 1980 and XZ69 caused typical lesions on detached rapeseed leaves (Fig. 1C). However, the average diameter of lesions caused by strain XZ69 was 2.40 cm, significantly smaller than the lesions caused by strain 1980 (3.08 cm) (Fig. 1D), suggesting that strain XZ69 was a less virulent strain of *S. sclerotiorum* compared to strain 1980. In order to explore the biocontrol potential of XZ69 against Sclerotinia stem rot, we assayed potential biocontrol by hypovirulent strain XZ69 of *S. sclerotiorum* by following the procedure illustrated in Fig. 1E. The results showed that pre-spraying with a mycelial suspension of strain XZ69 provided protection for the rapeseed seedlings against severe stem rot disease, indicating the potential of strain XZ69 for biological control against *S. sclerotiorum* infection (Fig. 1F).

### 3.2 Six mycoviruses co-infect strain XZ69 of *S. sclerotiorum*

**Fig. 2:**
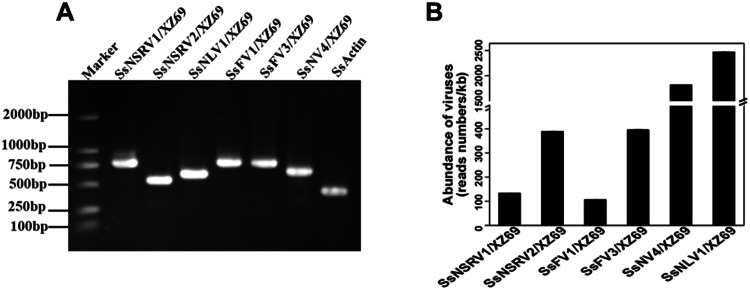
RT-PCR detection of mycoviruses and analysis of mycovirus abundance in strain XZ69. (A) Confirmation of mycovirus-related contigs assembled by Illumina sequencing of strain XZ69 using RT-PCR. Lane Marker, DL2000, DNA molecular weight marker (Code No: 3427Q; Company: TaKaRa, Dalian, China). (B) Abundance of mycoviruses in strain XZ69; Y axis represents the number of reads/kb.

To explore mycovirus composition, we conducted RNA-Seq analysis for strain XZ69. A total of 100,170,130 raw reads were generated through Illumina sequencing. After removing low-quality reads, 99,792,750 high-quality reads remained. These high-quality reads were compared with the *S. sclerotiorum* genome (GCF_000146945.2) to remove reads matching the host fungus, and the remaining reads were assembled into 14,244 contigs. Based on BLASTx results, only 0.2 % (24/14,244) of these contigs might be related to previously known viruses. Among these virus-related contigs, six that were longer than 1000 nt in length were selected, and sequences with more than 90 % identity to each other were de-duplicated. Through BLASTp comparison and RT-PCR detection, it was confirmed that strain XZ69 is co-infected with six mycoviruses (Fig. 2A), including four (+) ssRNA mycoviruses and two (-) ssRNA mycoviruses. Five of these mycoviruses shared more than 90 % identity to previously reported mycoviruses, and were provisionally named Sclerotinia sclerotiorum negative-stranded RNA virus 1/XZ69 (SsNSRV1/XZ69), SsNSRV2/XZ69, Sclerotinia sclerotiorum fusarivirus 1 (SsFV1/XZ69), SsFV3/XZ69, and Sclerotinia sclerotiorum narnavirus 4/XZ69 (SsNV4/XZ69). A contig exhibiting 60.4 % identity to the reported Monilinia narnavirus H, was named Sclerotinia sclerotiorum narna-like virus 1/XZ69 (SsNLV1/XZ69). Detailed information on the identified mycoviruses is shown in Table 1. The abundance analysis of the six mycoviruses in strain XZ69 revealed that SsNLV1/XZ69 was the most abundant, followed by SsNV4/XZ69 (Fig. 2B).

**Table 1:** The information of the six viruses in strain XZ69.

**Virus Name**	**Abbreviation**	**Length (nt)**	**Best match (BLASTx)**	**Sequence Identity (%)**	**GenBank Accession No**	**Genome Type**	**Family**
Sclerotinia sclerotiorum negative-stranded RNA virus 1/XZ69	SsNSRV1/XZ69 (PQ328748)	10,005	Sclerotinia sclerotiorum negative-stranded RNA virus 1-HC025	96.48 % (RdRp)	UMO80451.1	(-) ssRNA	*Mymonaviridae*
Sclerotinia sclerotiorum negative-stranded RNA virus 2/XZ69	SsNSRV2/XZ69 (PQ328749)	9608	Sclerotinia sclerotiorum negative-stranded RNA virus 2-A	92.45 % (RdRp)	AWY11014.1	(-) ssRNA	*Mymonaviridae*
Sclerotinia sclerotiorum fusarivirus 1/XZ69	SsFV1/XZ69 (PQ328750)	7658	Sclerotinia sclerotiorum fusarivirus 1	96.40 % (polyprotein)	YP_009143301.1	(+) ssRNA	*Fusariviridae*
Sclerotinia sclerotiorum fusarivirus 3/XZ69	SsFV3/XZ69 (PQ328747)	6413	Botrytis cinerea fusarivirus 8	97.80 % (RdRp)	UVT84595.1	(+) ssRNA	*Fusariviridae*
Sclerotinia sclerotiorum narnavirus 4/XZ69	SsNV4/XZ69 (PQ328751)	3106	Sclerotinia sclerotiorum narnavirus 4 RNA1	92.71 % (RdRp)	QZE12024.1	(+) ssRNA	*Narnaviridae*
Sclerotinia sclerotiorum narnavirus 4/XZ69	SsNV4/XZ69 (PQ328752)	2465	Sclerotinia sclerotiorum narnavirus 4 RNA2	86.54 %(Hp)	QZE12025.1	(+) ssRNA	*Narnaviridae*
Sclerotinia sclerotiorum narna-like virus 1 /XZ69	SsNLV1/XZ69 (PQ328746)	3521	Monilinia narnavirus H	60.35 % (RdRp)	QED42934.1	(+) ssRNA	Unclassified

### 3.3 SsNLV1/XZ69, a new monosegmented narna-like virus, phylogenetically related to viruses potentially infecting organisms across three kingdoms

**Fig. 3:**
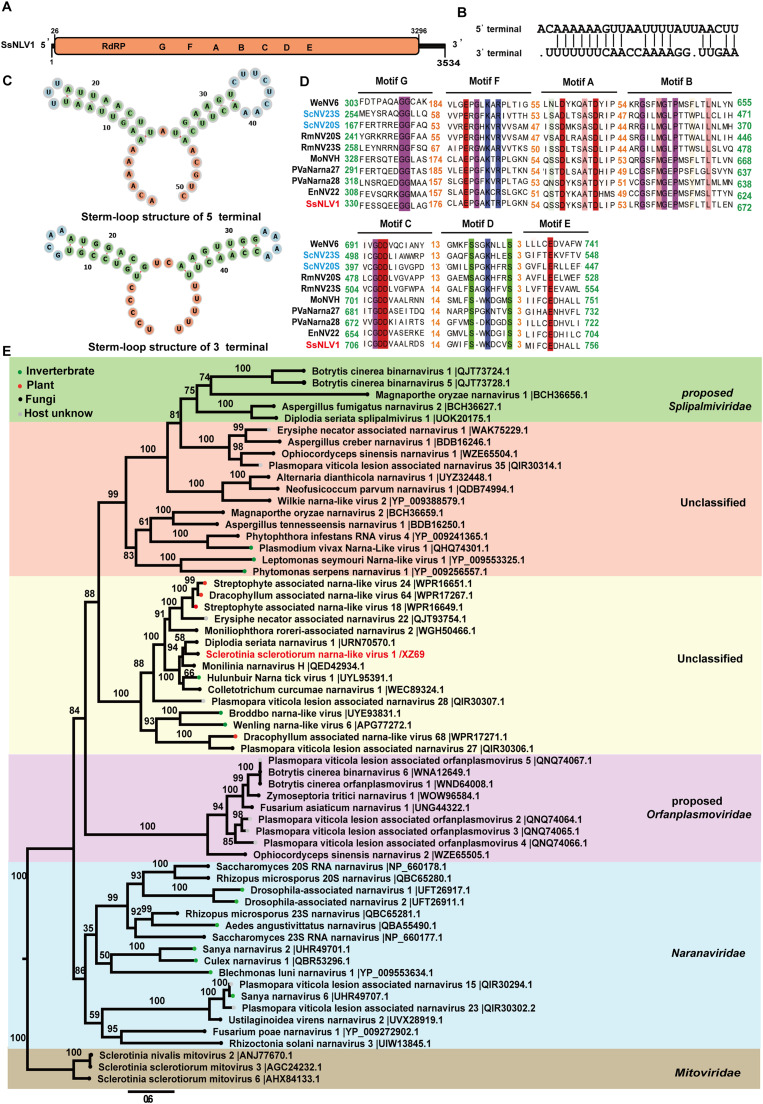
The genome organization, multiple alignments, and phylogenetic analysis of SsNLV1/XZ69. (A) Genomic organization of SsNLV1/XZ69, including 5′ UTR, 3′ UTR, and ORF regions. ORFs are indicated by rectangular boxes. (B) Multiple alignments of 5′-terminal and 3′-terminal sequences of the SsNLV1/XZ69 genomic segments. (C) The stem-loop structures of terminal sequences of SsNLV1/XZ69 genomic segments. Viral RNAs were predicted using the RNA folding form at the website (http://www.unafold.org/mfold/applications/rna-folding-form-v2.php). (D) Multiple alignment of the conserved RdRp domain of SsNLV1/XZ69 with other narna-like viruses. The names of viruses identified in this study are marked in red, previously described viruses are marked in blue, and viruses from the NCBI database are marked in black. GenBank accession numbers and virus names are listed in Table S2. Conserved amino acids are shown in different colors, and the numbers show the count of amino acids spaced between different conserved regions. Seven conserved motifs in the RdRp protein of narna-like viruses are marked above the sequence. (E) Phylogenetic analysis of SsNLV1/XZ69 and other selected narnavirus and narna-like viruses based on the RdRp using the Maximum Likelihood method with 1000 bootstrap replicates. Members of the family *Mitoviridae* were used as an outgroup. The names of viruses identified in this study are marked in red. The scale bar in the lower left corresponds to a genetic distance of 0.6. The virus names are followed by their registration numbers.

We obtained the full-length genome of SsNLV1 by terminal cloning. The genome of SsNLV1/XZ69 is comprised of 3534 nucleotides (nt) with a GC content of 46.9 % (Fig. 3A). SsNLV1/XZ69 contains a single ORF, which starts at nt 26 and ends with a UAG termination codon at nt 3296, encoding a 1090 aa RNA dependent RNA polymerase (RdRp) with a predicted molecular weight of approximately 123 kDa (Fig. 3A). SsNLV1/XZ69, along with Saccharomyces 20S RNA narnavirus (ScNV20S) and Saccharomyces 23S RNA narnavirus (ScNV23S), share similar genomic structural features, including a monosegmented genome and stem-loop structures at the 5′ and 3′ ends, but it lacks a poly(A) tail at the 3′ end (Fig. 3A and C). However, unlike ScNV20S and ScNV23S, SsNLV1/XZ69 does not have GGGGC at the 5′ end complementary to the 3′ end, and it exhibits partial complementation between its 5′ and 3′ ends (Fig. 3B).

The RdRp of SsNLV1/XZ69 shares 60.4 % and 39.3 % identities with that of Monilinia narnavirus H (MoNVH) and Erysiphe necator associated narnavirus 22 (EnNV22), respectively, both of which were isolated from filamentous fungi. SsNLV1/XZ69 shares 31.9 % and 30.1 % identities with Plasmopara viticola lesion associated narnavirus 28 (PVaNarna28) and PVaNarna27, respectively, both of which potentially infect oomycetes. Additionally, the RdRp of SsNLV1/XZ69 shares 29.9 %, 28.9 %, and 25.7 % identities with Wenling narna-like virus 6 (WeNV6), Sherlock virus, and Aedes japonicus narnavirus 1 (AjNV1), respectively, all of which infect insects. To determine the evolutionary relationships of SsNLV1/XZ69 with other reported viruses, multiple alignments were performed based on RdRp sequences. The results showed that the RdRp of SsNLV1/XZ69 contained seven typical conserved motifs (G, F, A, B, C, D, and E) (Fig. 3D). A maximum likelihood phylogenetic tree revealed that SsNLV1/XZ69 has a close relationship to MoNVH and forms a distinct branch with unclassified viruses associated with fungi, oomycetes, plants, and insects (Fig. 3E), supporting the establishment of a new family to accommodate these unclassified viruses.

### 3.4 Molecular characterization of SsFV3/XZ69 reveals the potential cross-genus transmission

**Fig. 4:**
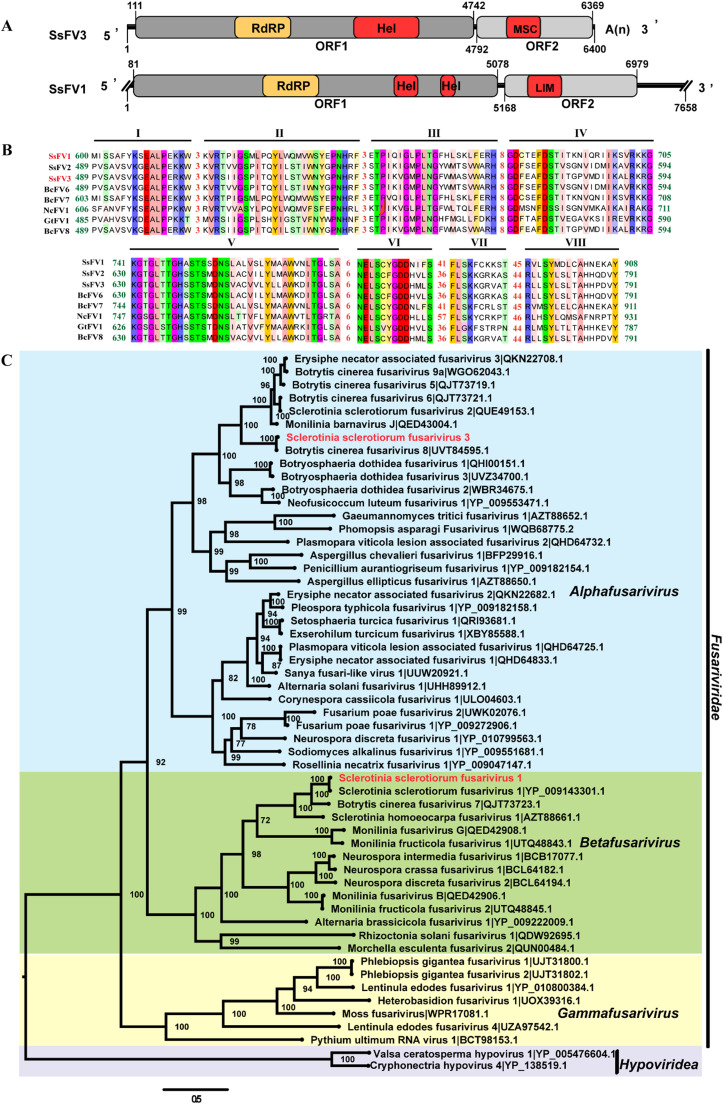
Genomic structure and phylogenetic tree of SsFV3/XZ69. (A) Genomic organization of SsFV3/XZ69 and SsFV1/XZ69 (upper panels). Double oblique lines at the ends represent unverified or incomplete genomes. Rectangular boxes represent ORFs. Two conserved domains, RdRp and helicase (Hel), are indicated in the polyprotein encoded by ORF1. SsFV3/XZ69 has a poly(A) structure inside the 3′-UTR, excluding the poly(A) tail. The genomic structure of SsFV3 is similar to that of the reported SsFV1/XZ69. (B) Multiple alignment of the polyprotein of SsFV3/XZ69 and SsFV1/XZ69. The names of viruses identified in this study are marked in red, and NCBI database viruses are marked in black. GenBank accession numbers and virus names are listed in Table S2. Conserved amino acids are shown in different colors, with numbers indicating the spacing of amino acids between conserved regions. Eight conserved motifs in the polyprotein are marked above the sequence. (D) Maximum Likelihood phylogenetic tree constructed based on multiple alignment of the conserved RdRp domain of selected viruses with 1000 bootstrap replicates. Virus names are followed by their accession numbers. Viruses within *Hypoviridae* were selected as the outgroup.

We also obtained the full-length genome of SsFV3/XZ69 within the family *Fusariviridae*. The genome of SsFV3/XZ69 is comprised of 6400 nt with a GC content of 35.4 %. SsFV3/XZ69 contains two non-overlapping ORFs (Fig. 4A). ORF1 starts at nt 111 and ends with a UAG termination codon at nt 4742, encoding a 1542 amino acids polyprotein with a predicted molecular weight of approximately 174 kDa, which includes three transmembrane domains (1–205 aa). ORF2 starts at nt 4792 and ends with a UAG termination codon at nt 6369, encoding a 525 aa hypothetical protein with a predicted molecular weight of approximately 58 kDa (Figs. 4A and S1). SsFV1/XZ69 also contains two non-overlapping ORFs (Fig. 4A). ORF1 encodes a polyprotein consisting of 1665 amino acids, predicted to have one RdRp domain and two helicase domains, and also features a transmembrane domain (Figs. 4A and S1). SsFV3/XZ69 shows 87.9 % identity with Botrytis cinerea fusarivirus 8 (BcFV8) at the nucleotide level. The polyprotein encoded by SsFV3/XZ69 shares 97 %, 58 %, and 57 % identities with the RdRp of BcFV8, Erysiphe necator associated fusarivirus 3 (EnFV3), and BcFV5, respectively. Motif prediction analysis indicated that the protein encoded by SsFV3/XZ69 ORF1 contains two conserved domains: an RdRp and a helicase (Fig. 4A). SsFV3/XZ69 ORF2-encoded protein has a conserved SMC (structural maintenance of chromosomes) domain, which has been detected in BcFV8, Pleospora typhicola fusarivirus 1 (PtFV1), Plasmopara viticola lesion-associated fusarivirus 1 (PvlaFV1), and PvlaFV3. This protein has 97 %, 32 %, and 32 % aa identities with BcFV8, EnFV3, and BcFV5, respectively. Another fusarivirus, SsFV1/XZ69, ORF1-encoded and ORF2-encoded protein show 68.1 % and 56.2 % identity with Botrytis cinerea fusarivirus 7 ORF1-encoded protein and ORF2-encoded protein, respectively. To explore the evolutionary relationship of SsFV3/XZ69 and SsFV1/XZ69 with other reported fusariviruses, multiple alignments revealed that eight conserved motifs are detected in the RdRp domain of SsFV3/XZ69 and of SsFV1/XZ69 (Fig. 4B). A Maximum Likelihood phylogenetic tree revealed that SsFV3/XZ69 and SsFV1/XZ69 belong to the genera *Alphafusarivirus* and *Betafusarivirus* within the family *Fusariviridae*, respectively (Fig. 4C). In conclusion, both SsFV3/XZ69 and BcFV8 have similar genomic structures as well as conserved structural domains. The proteins encoded by ORF1 and ORF2 are 97 % identical. Evolutionary analysis of the closest relatives of SsFV3/XZ69 and BcFV8 suggests the possibility of cross-genus transmission between *S. sclerotiorum* and *B. cinerea*.

### 3.5 Hypovirulence associated with SsFV3/XZ69, SsNSRV1/XZ69 and SsNSRV2/XZ69

**Fig. 5:**
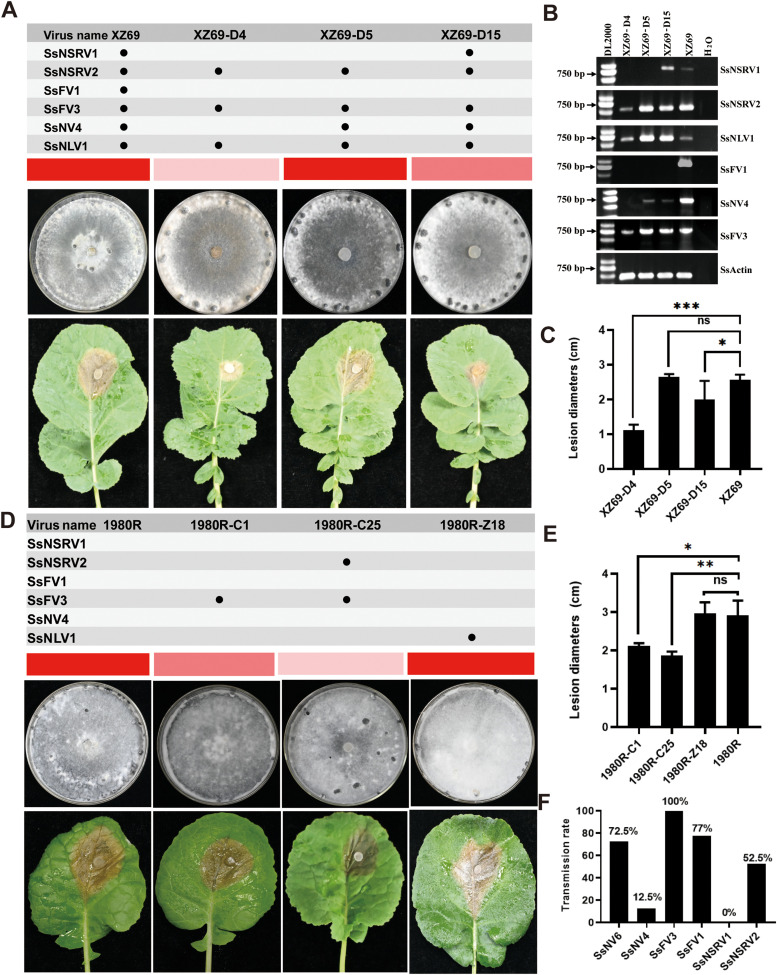
SsFV3/XZ69, SsNSRV1/XZ69, and SsNSRV2/XZ69 are associated with hypovirulence in *S. sclerotiorum*. (A) Colony morphology of protoplast regeneration strains derived from strain XZ69 on PDA (7 dpi, 20 °C) and virulence assay on detached rapeseed leaves (2 dpi, 20 °C). Red entries above the colony morphology correspond to pathogenicity, and light red color indicates weak pathogenicity. Virus species contained in each strain are shown above the red entries. (B) RT-PCR detection of mycovirus content using mycovirus-specific primers (Table S1). (C) Statistical analysis of lesion diameters of inoculated XZ69, XZ69-D4, XZ69-D5, and XZ69-D15 leaves, with error bars representing the standard deviation calculated from three replicates. Significant differences at be consistent *p* < 0.05 were determined using *t*-test. (D) Colony morphology of 1980R and its three newly obtained mycovirus-infected strains on PDA (12 dpi, 20 °C) and virulence assay on detached rapeseed leaves (2 dpi, 20 °C). Red entries above the colony morphology correspond to pathogenicity, and light red color indicates weak pathogenicity. Virus species contained in each strain are shown above the red entries. (E) Statistical analysis of lesion diameters on detached rapeseed leaves by 1980R background strains carrying the mycoviruses, with error bars representing the standard deviation calculated from three replicates. Significant differences at *p* < 0.05 were determined using *t*-test. (F) Horizontal transmission rate of mycoviruses from strain XZ69 (donor) to strain 1980R (recipient).

To investigate which mycovirus is a hypovirulent factor in strain XZ69 of *S. sclerotiorum*, we obtained three protoplast regeneration strains XZ69-D4, XZ69-D5, and XZ69-D15 with different mycovirus combinations. XZ69-D4 is co-infected by SsNSRV2/XZ69, SsFV3/XZ69, and SsNLV1/XZ69. XZ69-D5 harbors SsNSRV2/XZ69, SsFV3/XZ69, SsNV4/XZ69, and SsNLV1/XZ69. XZ69-D15 is confirmed to be co-infected by five mycoviruses (SsNSRV1/XZ69, SsNSRV2/XZ69, SsFV3/XZ69, SsNV4/XZ69, and SsNLV1/XZ69) (Fig. 5A and B). Strains XZ69-D4 and XZ69-D15 cause smaller lesions on the detached leaves compared to strain XZ69; however, strain XZ69-D5 has a similar lesion size compared to strain XZ69 (Fig. 5A and C), suggesting that SsNSRV2/XZ69, SsFV3/XZ69, SsNLV1/XZ69, and SsNSRV1/XZ69 may be associated with hypovirulence in *S. sclerotiorum* (Fig. 5A and C).

To further investigate mycoviruses directly associated with hypovirulence, we conducted mycovirus horizontal transmission and mycoviral RNA transfection assays. Strain XZ69 was dual-cultured with strain 1980R. When hyphae of these two strains were in contact for 3 days; then hyphae at colony margins of strain 1980R were transferred to fresh PDA containing hygromycin B (50 μg/mL). We obtained new strains named 1980R-C1 and 1980R-C25. 1980R-C1 harbors SsFV3/XZ69, while 1980R-C25 is co-infected by SsFV3 and SsNSRV2/XZ69 (Figs. 5D and S2). Additionally, total RNA from XZ69-D4 co-infected by SsNSRV2/XZ69, SsFV3/XZ69, and SsNLV1/XZ69 was used to transfect protoplasts of strain 1980R; the transfectant (strain 1980R-Z18) harboring SsNLV1/XZ69 was successfully obtained (Figs. 5D and S3), demonstrating that the purified RNA of SsNLV1/XZ69 possesses infective activity.

Strains 1980R-C1 and 1980R-C25 showed lower virulence on detached leaves compared to strain 1980R, while 1980R-Z18 has a similar virulence to strain 1980R (Fig. 5D and E). Thus, we concluded that SsFV3/XZ69 and SsNSRV2/XZ69 could be factors for hypovirulence in strain of *S. sclerotiorum*. RT-PCR of 40 strains newly obtained using dual-culture between strains XZ69 and 1980R revealed that SsFV3/XZ69 achieved 100 % horizontal transmission efficiency (Fig. 5F). Therefore, the mycoviruses that contribute to hypovirulence in XZ69 could be SsFV3/XZ69, SsNSRV2/XZ69 and SsNSRV1/XZ69.

## 4 Discussion

Hypovirulence-associated mycoviruses have emerged as a promising form of biocontrol to manage diseases (Wagemans et al., 2022; Xie and Jiang, 2024). In this study, we identified a hypovirulent strain XZ69 co-infected by six mycoviruses in three families. Pre-spraying rapeseed seedlings with a mycelial suspension of strain XZ69 can effectively protect plants, highlighting its potential for biocontrol against diseases caused by *S. sclerotiorum*. SsNLV1/XZ69 clustered with a group of narna-like viruses infecting fungi, plants, and animals to form a separate evolutionary branch, supporting the establishment of a new family. SsFV3/XZ69 may have the potential for cross-genus transmission between *S. sclerotiorum* and *B. cinerea*. In addition, we found that SsFV3/XZ69, in combination with SsNSRV1/XZ69 and SsNSRV2/XZ69, is potential hypovirulent factors in strain XZ69.

The main hypovirulent factors for strain XZ69 were explored through multiple methods, including virome analysis, mycovirus elimination, and mycovirus transmission. Similar to previous reports in *S. sclerotiorum* (Hai et al., 2022; Mu et al., 2021), mycovirus infection is responsible for the hypovirulent phenotypes of strain XZ69. Three mycoviruses, including SsFV3/XZ69, SsNSRV1/XZ69, and SsNSRV2/XZ69, are potentially associated with hypovirulence in *S. sclerotiorum*. However, direct evidence linking specific viruses to virulence remains limited due to challenges in obtaining fully cured strains or single-virus isolates in the XZ69 background. SsFV1/XZ69 is not associated with hypovirulence, which is consistent with previous reports for strain JMTJ14 infected by SsFV1/JMTJ14 (Liu et al., 2015). However, BcFV8 is responsible for hypovirulence in *B. cinerea* isolate Kst14a (Ahmed et al., 2024). In this study, we also confirmed that SsFV3/XZ69 is a potential hypovirulent factor in *S. sclerotiorum*. To date, the direct evidence linking SsNSRV1/XZ69 and SsNSRV2/XZ69 to hypovirulence in strain XZ69 is lacking, and strains exclusively infected with these mycoviruses have not been successfully isolated. Nevertheless, SsNSRV1/AH98 infecting the strain AH98 of *S. sclerotiorum* has been shown to be associated with hypovirulence (Liu et al., 2014), supporting the evidence that SsNSRV1/XZ69 is a potential hypovirulent factor. SsNLV1/XZ69 did not contribute to hypovirulence, and further research is needed to examine possible reasons. For example, the protein of SsNLV1/XZ69 is unable to effectively interact with the key pathogenic factors of *S. sclerotiorum*, or it does not significantly alter the host's metabolic pathways and genes expression. An ultimate goal of research on hypovirulent strains is to apply them in the field and utilize their biological value.

Mycovirus-mediated hypovirulent strains show potential biocontrol of phytopathogenic fungal diseases via multiple biocontrol mechanisms. Firstly, hypovirulence-associated mycoviruses should be able to efficiently transmit in fungal populations. The classic example is that CHV1 has been successfully applied to control chestnut blight in Europe (Rigling and Prospero, 2018). Hypovirulent-associated virus SsFV3/XZ69 in strain XZ69 spreads horizontally into strain 1980R with a transmission rate of up to 100 %, which may be one of the reasons why XZ69 has shown efficacious results in biological control trials. Secondly, mycovirus-mediated hypovirulent strains may be able to activate plant systemic resistance for disease control. For example, *Leptosphaeria biglobosa* infected by Leptosphaeria biglobosa quadrivirus 1 can induce systemic resistance in *Brassica napus* against *L. maculans* infection (Shah et al., 2020). In our study, we confirmed that strain XZ69 shows potential biocontrol to combat *S. sclerotiorum*. However, whether strain XZ69 does trigger systemic resistance in rapeseed plants and spread mycoviruses to virulent strains needs to be further explored.

We first report a monosegmented narna-like mycovirus in *S. sclerotiorum*. Phylogenetic analyses indicated that SsNLV1/XZ69 does not belong to known families, and forms an independent evolutionary branch with 14 other reported viruses, but they lack complete genomes. This unique cluster is phylogenetically positioned between the two proposed families, "*Orfanplasmoviridae"* and "*Splipalmiviridae"* (Chiapello et al., 2020, Chiba et al., 2021, Jia et al., 2024). Therefore, we propose to establish a new family to accommodate these viruses. There are two points worth noting. The first point is that SsNLV1/XZ69 is the only virus with a full-length genome among all 15 reported viruses tentatively assigned to this new proposed family. Therefore, more complete genomes of this virus group need to be sequenced, and this will contribute to a better understanding of virus structure and evolution within this newly proposed family. Another point is that SsNLV1/XZ69 and its phylogenetically related viruses are associated with diverse hosts, including invertebrates, plants, oomycetes, and fungi. However, these viruses associated with invertebrates and plants were mostly obtained by collecting natural samples and using metatranscriptomic sequencing (French et al., 2023; Shi et al., 2016), and their true hosts have not yet been confirmed. Additionally, to adapt to the environment, plant viruses have chosen an evolutionary strategy that ranges from generalization to specialization (Lefeuvre et al., 2019), and the evolution of mycoviruses may also involve this kind of evolutionary approach.

Mycoviruses are generally considered to have limited host range (García-Pedrajas et al., 2019). However, the phenomenon that phylogenetically distant fungal species can be infected by the same or phylogenetically close mycovirus has been frequently observed (Xie and Jiang, 2014). For example, Trichoderma koningiopsis totivirus 1 (TkTV1/Mg10) can naturally occur in *Trichoderma koningiopsis* (Hypocreaceae) and *Clonostachys rosea* (Bionectriaceae), which belong to different fungal families (Khalifa and MacDiarmid, 2019). Ophiostoma mitovirus 3a (OMV3a) and OMV3b have been detected in three different fungi: *Ophiostoma novoulmi* (Ophiostomataceae), *Sclerotinia homoeocarpa* (Rutstroemiaceae), and *B. cinerea* (Sclerotiniaceae) (Deng et al., 2003; Wu et al., 2010). Leptosphaeria biglobosa botybirnavirus 1 (LbBV1) is detected in *Leptosphaeria biglobosa* (Leptosphaeriaceae) and *B. cinerea* (Sclerotiniaceae), and there is experimental evidence of cross-genus transmission of LbBV1 between *L. biglobosa* and *B. cinerea* on rapeseed plants (Deng et al., 2022). In addition, there are some viruses in *B. cinerea* that are also present in *S. sclerotiorum*, such as Botrytis porri RNA virus 1, Botrytis virus F, and Botrytis porri botybirnavirus 1 (Jia et al., 2021; Mu et al., 2018; Ruiz-Padilla et al., 2021). The possible reasons regarding cross-species/genus transmission of mycoviruses are the existence of intersecting fungal host ranges and sharing the same ecological niche. In this study, SsFV3/XZ69 was found to be shared in the viromes of *S. sclerotiorum* and *B. cinerea*, suggesting the possibility of cross-genus transmission. However, when *B. cinerea* was co-inoculated with strain XZ69 in living rapeseed plants following previously reported methods (Wu et al., 2007), mycoviruses in strain XZ69 failed to transmit to the *B. cinerea* isolates. The primary reason for the failure of horizontal transmission of SsFV3 may be due to the vegetative incompatibility between the *S. sclerotiorum* strain XZ69 and the *B. cinerea* strain B05.10, which impedes the spread and replication of the mycovirus.

In summary, our study identified a hypovirulent strain XZ69, that is co-infected by six mycoviruses. SsNLV1/XZ69 is a novel single-segmented narna-like virus, and we propose that a new virus family should be established to accommodate these unclassified SsNLV1-related viruses infecting organisms across multiple kingdoms. Additionally, we demonstrated that SsFV3/XZ69, SsNSRV1/XZ69, and SsNSRV2/XZ69 are associated with the hypovirulent phenotype of *S. sclerotiorum*. This study increases mycovirus resources for biological control of *S. sclerotiorum*, provides transmission characteristics, further demonstrates the prevalence of complex infections, and provides new material for virus interactions.

## CRediT authorship contribution statement

**Lixia Gao:** Writing – original draft, Validation, Methodology, Formal analysis, Data curation. **Weimeng Li:** Validation, Methodology, Formal analysis. **Jichun Jia:** Methodology, Formal analysis, Data curation. **Jiasen Cheng:** Supervision, Resources, Project administration. **Yanping Fu:** Supervision, Resources, Methodology. **Xueqiong Xiao:** Writing – review & editing, Supervision, Project administration, Methodology. **Qing Cai:** Supervision, Resources, Formal analysis. **Yang Lin:** Resources, Methodology. **Tao Chen:** Resources. **Bo Li:** Supervision, Resources. **Xiao Yu:** Supervision, Resources. **Tom Hsiang:** Writing – review & editing, Supervision. **Daohong Jiang:** Writing – review & editing, Supervision, Resources, Project administration, Funding acquisition. **Jiatao Xie:** Writing – review & editing, Supervision, Resources, Project administration, Methodology, Funding acquisition.

## Declaration of competing interest

The authors declare no conflict of interests.

## Data Availability

The authors do not have permission to share data.

## Appendix Supplementary materials

Image, application 1
