# Supplementary material for: Exploration of mycovirus composition in a hypovirulent strain of Sclerotinia sclerotiorum potentially uncovers mycovirus cross-taxa transmission
Source: Virus Res. 2025 Feb 26;354:199552. doi: 10.1016/j.virusres.2025.199552 (PMC11925586; doi:10.1016/j.virusres.2025.199552)
Supplement: Supplementary file 1 [file mmc1.doc]

**Supplementary Material**

**Table S1** Primers sequence information involved in this study.

| **Num.** | **Primer Name** | **Primer Sequence (5'-3')** | **Application** | **Product size(bp)** |
| --- | --- | --- | --- | --- |
| 1 | SsNSRV1-R | TTTCGTCTTCGCTCCCTAC | Detection for SsNSRV1/XZ69 | 786 |
|  | SsNSRV1-F | CTGTATTGAGTATTGAACCGCC |  |  |
| 2 | SsNSRV2-R | GTGATAGATGGGACTACCGTC | Detection for SsNSRV2/XZ69 | 547 |
|  | SsNSRV2-F | GGGTCTTGATTCCAACGAC |  |  |
| 3 | SsFV3-R | GAATAATGCTTTGTCACCCG | Detection for SsFV3/XZ69 | 755 |
|  | SsFV3-F | TCATAGTGGCTGCCCTACTC |  |  |
| 4 | SsNLV1-F | GACAAGGCTCTCCCAAATAC | Detection for SsNLV1/XZ69 | 609 |
|  | SsNLV1-R | AGGGTGTTCCTGTTCAAGG |  |  |
| 5 | SsNV4-F | ATTCTTCCCATAGCAGCCG | Detection for SsNV4/XZ69 | 643 |
|  | SsNV4-R | TGACACGCTCGGTTCTAAGG |  |  |
| 6 | SsFV1-F | TTCTCATCTCCTCAGGGTGG | Detection for SsFV1/XZ69 | 771 |
|  | SsFV1-R | AGTCACTGTTGTCATCACTCCG |  |  |
| 7 | SsActin-F | CTGGAAGATTGACTGGCGGTTTG | Endogenous reference gene | 419 |
|  | SsActin-R | AGCACCAGAGGAGCACCCAGTTT |  |  |
| 8 | SsFV3-3-F2 | ATAAGCGTACCAAAACCAGC | SsFV3 3, terminal cloning | / |
|  | SsFV3-3-F3 | CAAGGTTTCGGCCACAACTG |  |  |
| 9 | SsFV3-5-F2 | GAATTGGCCATATTGCTGCT | SsFV3 5, terminal cloning | / |
|  | SsFV3-5-F3 | GAAACCACCAAAACCGTCCG |  |  |
| 10 | SsNLV1-3F2 | CAGTTGGCCTCTCTGGGTAA | SsNLV1 3, terminal cloning | / |
|  | SsNLV1-3F3 | AACATGGCACGTCAAGTTCG |  |  |
| 11 | SsNLV1-5F2 | CCGTTTATGTGTCCCTTGCG | SsNLV1 5, terminal cloning | / |
|  | SsNLV1-5F3 | AACCCAGCCTCTCCAATACG |  |  |
| 12 | PC2 | CCGAATTCCCGGGATCC | Terminal cloning | / |
|  | PC3-T7 loop | GGATCCCGGGAATTCGGTAATACGACTCACTATATTTTTATAGTGAGTCGTATTA |  |  |

**Table S2** Viral information used for multiple sequence alignment.

| Number | Virus name | Accession no. | Abbreviation |
| --- | --- | --- | --- |
| 1 | Monilinia narnavirus H | QED42934.1 | MoNVH |
| 2 | Erysiphe necator associated narnavirus 22 | QJT93754.1 | EnNV22 |
| 3 | Plasmopara viticola lesion associated narnavirus 28 | QIR30307.1 | PVaNarna28 |
| 4 | Wenling narna-like virus 6 | APG77272.1 | WeNV6 |
| 5 | Plasmopara viticola lesion associated narnavirus 27 | QIR30306.1 | PVaNarna27 |
| 6 | Saccharomyces 23S RNA narnavirus | NP_660177.1 | SsNV23S |
| 7 | Rhizopus microsporus 23S narnavirus | QBC65281.1 | RmNV23S |
| 8 | Saccharomyces 20S RNA narnavirus | NP_660178.1 | SsNV20S |
| 9 | Rhizopus microsporus 20S narnavirus | QBC65280.1 | RmNV20S |
| 10 | Sclerotinia sclerotiorum narna-like virus 1 | XHP99729.1 | SsNLV1 |
| 11 | Sclerotinia sclerotiorum fusarivirus 1 | XHP99743.1 | SsFV1 |
| 12 | Sclerotinia sclerotiorum fusarivirus 2 | QUE49153.1 | SsFV2 |
| 13 | Sclerotinia sclerotiorum fusarivirus 3 | XHP99730.1 | SsFV3 |
| 14 | Botrytis cinerea fusarivirus 6 | QJT73721.1 | BcFV6 |
| 15 | Botrytis cinerea fusarivirus 7 | QJT73723.1 | BcFV7 |
| 16 | Botrytis cinerea fusarivirus 8 | UVT84595.1 | BcFV8 |
| 17 | Neurospora crassa fusarivirus 1 | BCL64182.1 | NcFV1 |
| 18 | Gaeumannomyces tritici fusarivirus 1 | AZT88652.1 | GtFV1 |


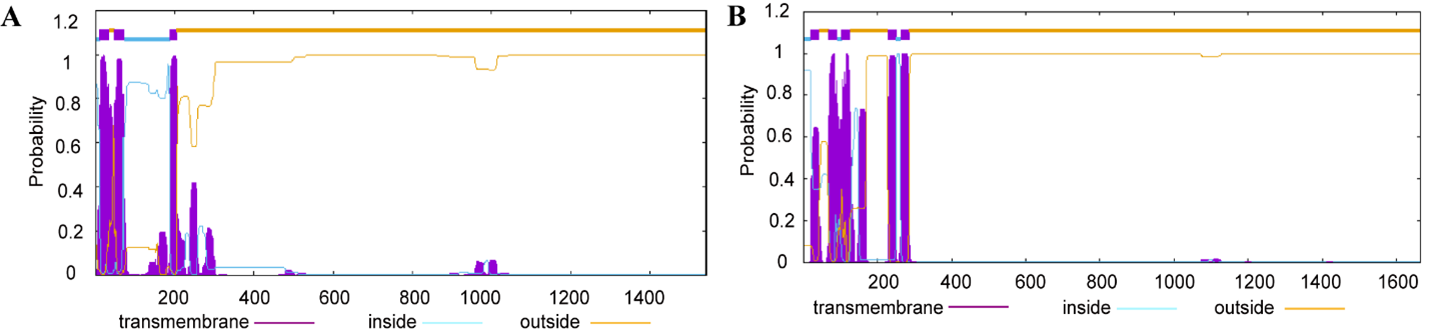


**Figure S1.** Analysis of transmembrane domains in the RdRp protein of SsFV1 and SsFV3. (A) Predicting transmembrane domains of the RdRp protein of SsFV1 on the web. (B) Predicting transmembrane domains of the RdRp protein sequence of SsFV3 on the web. The web address mentioned above is <https://services.healthtech.dtu.dk/services/TMHMM-2.0/>.


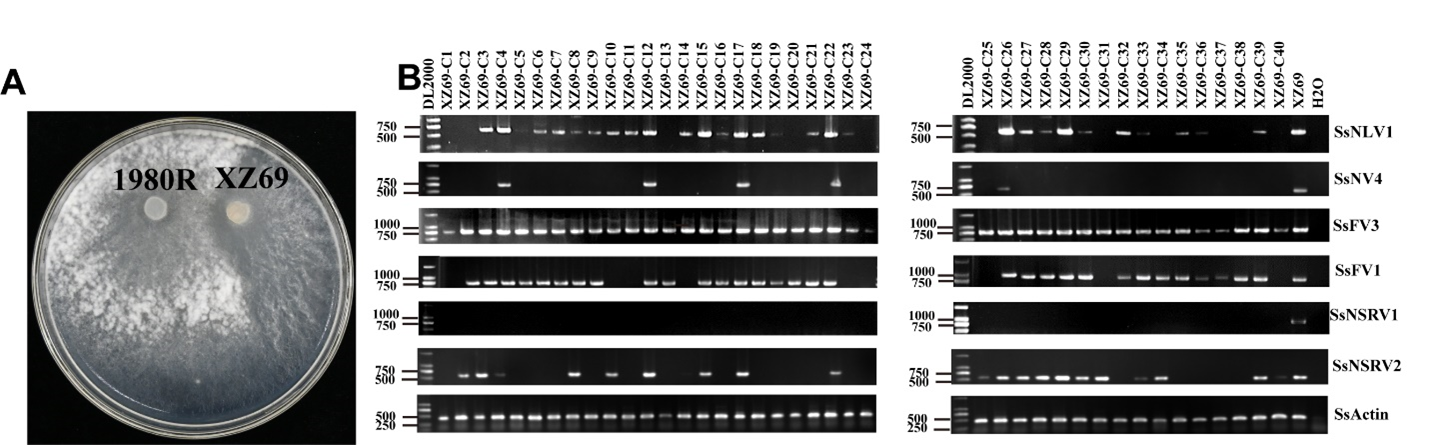


**Figure S2.** Viral detection in horizontally transmitted isolates. (A) Schematic diagram of horizontal transmission (Donor strain: XZ69; Recipient strain: 1980; At 20 °C for 72 h). (B) RT-PCR confirmation of strains transfected with viruses of strain XZ69. Lane Marker, DL2000, DNA molecular weight marker (Code No: 3427Q; Company: Takara Dalian, China); Mycovirus-specific primers were designed from sequenced contigs to detect mycoviruses in strain XZ69 via PCR amplification (Table S1).


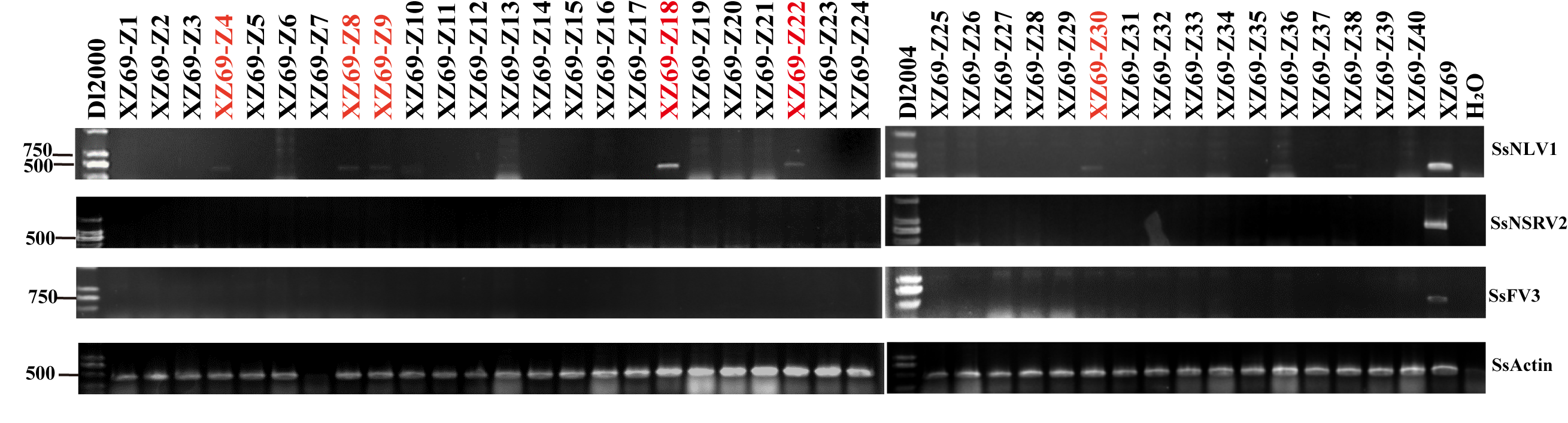


**Figure S3.** RT-PCR identification of the virus carried by the candidate transfectants. Lane Marker, DL2000, DNA molecular weight marker (Code No: 3427Q; Company: Takara Dalian, China); Mycovirus-specific primers were designed from assembled contigs to detect mycoviruses in strain XZ69 via PCR amplification (Table S1).
